# Supplementary material for: Pneumococcal meningitis and endotoxemia: A cross-sectional clinical study
Source: PLoS One. 2025 May 21;20(5):e0324153. doi: 10.1371/journal.pone.0324153 (PMC12094716; doi:10.1371/journal.pone.0324153)
Supplement: Attachment — Submitted filename: response to reviewers.docx [file pone.0324153.s003.docx]

**Response to Reviewer #1:**

We would like to thank the reviewer 1 for all the comments and advice that we carefully followed in order to improve the manuscript

1. **"Where is intestinal Fatty Acid Binding Protein found, what functions does it perform, what does its height indicate. Which diseases are valuable in measuring intestinal Fatty Acid Binding Protein? Is there a defined normal value for intestinal Fatty Acid Binding Protein? It is recommended to specify in the introduction section."**
   - **Response**: We have added detailed information in the Introduction section regarding the location, functions, and clinical relevance of iFABP.

*"* There are biomarkers capable of reflecting this intestinal dysfunction. For example, intestinal fatty acid binding protein (I-FABP) is a marker of intestinal damage (ID), released into circulation when the integrity of enterocyte membranes in the small intestine is compromised. Primarily expressed in enterocytes, I-FABP plays a critical role in fatty acid transport. Elevated plasma I-FABP levels indicate intestinal mucosal injury or ischemia and are used as a biomarker in conditions like inflammatory bowel disease and acute intestinal ischemia. In healthy individuals, plasma I-FABP levels typically range between 200-600 pg/ml (1,2)*"*

2. **"How do you explain the finding of higher intestinal Fatty Acid Binding Protein levels in healthy individuals, contrary to the hypothesis of your study?"**
   - **Response**: In the Discussion section, we addressed the point that “we observed that the surrogate marker of gut leakage, namely iFABP concentrations, were actually lower in meningitis patients than in healthy volunteers. This finding has also been reported in the context of COVID-19 and pneumococcal pneumonia (3,4)” Other studies have shown higher concentrations of iFABP in healthy volunteers compared to patients with mesenteric ischemia, a condition associated with intestinal injury (5). While one might hypothesize an analytical error, the concentrations obtained in our healthy volunteers (432 (248-651) pg/ml) fall within the known average ranges (i.e., 200–600 pg/ml). The question of differences between the assay tools used can also be raised. Nevertheless, some authors have validated these observations using multiple techniques (5). iFABP levels in some healthy individuals may reflect non-pathological individual variations, such as oxidative stress or age-related factors. However, age was balanced between the two groups, and no underlying diseases causing intestinal ischemia were identified in healthy individuals. Finally, the timing of measurements could explain some discrepancies in results between studies. iFABP is an early marker of intestinal epithelial damage; however, its concentration may decrease as the damage resolves, which can result in variability depending on the timing of sample collection. In our study, samples from meningitis patients were collected as soon as the pneumococcal strain was identified, approximately two days after admission. We cannot rule out the possibility of an earlier increase in iFABP levels occurring immediately upon admission, followed by a subsequent decrease.

“In addition, we observed that the surrogate marker of gut leakage, namely iFABP concentrations, were actually lower in meningitis patients than in healthy volunteers. This has also been reported in the setting of COVID-19 and pneumococcal pneumonia (9,13). Other studies have also demonstrated higher concentrations of iFABP in healthy volunteers compared to patients with mesenteric ischemia, a condition well-known for its association with intestinal injury (14). Additionally, no underlying diseases causing intestinal ischemia were identified in healthy individuals. However, these discrepancies between studies could be attributed to the different tools used to measure iFABP, or the timing of the iFABP measurement. In our study, sample from meningitis patients were collected as soon as the pneumococcal strain was identified, approximately two days after admission. We cannot rule out the possibility of an earlier increase in iFABP levels occurring immediately upon admission, followed by a subsequent decrease, as iFABP concentrations may decline with the resolution of intestinal damage.”

3. **"Are you sure that there is no disease that causes intestinal ischemia in individuals defined as healthy individuals? If you are sure, please state this in the material method."**
   - **Response**: We updated the Methods section to specify : “Comorbid conditions, including those that could lead to intestinal ischemia, were rigorously assessed.” And in the results section : “and 34 HV, none of whom had conditions predisposing to intestinal ischemia.”

**Response to Reviewer #2:**

1. **General comment** – This is a very interesting research question that aims to define a role, if any, of LPS in the systemic inflammatory response during pneumococcal meningitis. The study is well written and diligently conducted. The results are significant and relevant, adding good information for understanding the pathophysiology of this disease. There are some points the authors should review, as follows
   - **Response**: We thank the Reviewer 2 for these positive and kind comments.
2. **"In Introduction section correct, in Line 3, Neisseria meningitidis (N. meningitidis)"**
   - **Response**: The requested correction has been made in the Introduction section.
3. **"Which was the time frame for including healthy volunteers? Describe it in the manuscript."**
   - **Response**: We added to the Methods section that healthy volunteers were included between April and June 2022.
4. **"The dried extract extracts - remove the duplicate word"**
   - **Response**: The typographical error has been corrected.
5. **"** **Then, we observed a difference between patients and volunteers, considering immunocompromised status, which may introduce a potential bias in the interpretation of plasma endotoxin concentrations. Since immunocompromised patients are susceptible to gut translocation, this can nevertheless be considered as a conservative bias”- I stopped at this sentence. I read it and reread it several times, but I didn't really understand what you meant by it. Would you please rephrase it? The difference observed for biomarkers was expected ”**
   - **Response**: This sentence has been revised for clarity:

*"**We observed a difference between patients and healthy volunteers due to the immunocompromised status of some patients, which could bias the interpretation of plasma endotoxin concentrations. However, as immunocompromised patients are more susceptible to bacterial translocation, this bias can be considered conservative, and not an issue to interpret our data as LPS concentrations were not increased in patients compared to healthy volunteers."*
